# The Transcriptional Roles of ALK Fusion Proteins in Tumorigenesis

**DOI:** 10.3390/cancers11081074

**Published:** 2019-07-30

**Authors:** Stephen P. Ducray, Karthikraj Natarajan, Gavin D. Garland, Suzanne D. Turner, Gerda Egger

**Affiliations:** 1 Division of Cellular and Molecular Pathology, Department of Pathology, University of Cambridge, Cambridge CB20QQ, UK; 2 Department of Pathology, Medical University Vienna, 1090 Vienna, Austria; 3 Ludwig Boltzmann Institute Applied Diagnostics, 1090 Vienna, Austria

**Keywords:** ALK, ALCL, NPM-ALK, EML4-ALK, NSCLC, *ALK*-translocation proteins, epigenetics

## Abstract

Anaplastic lymphoma kinase (ALK) is a tyrosine kinase involved in neuronal and gut development. Initially discovered in T cell lymphoma, ALK is frequently affected in diverse cancers by oncogenic translocations. These translocations involve different fusion partners that facilitate multimerisation and autophosphorylation of ALK, resulting in a constitutively active tyrosine kinase with oncogenic potential. ALK fusion proteins are involved in diverse cellular signalling pathways, such as Ras/extracellular signal-regulated kinase (ERK), phosphatidylinositol 3-kinase (PI3K)/Akt and Janus protein tyrosine kinase (JAK)/STAT. Furthermore, ALK is implicated in epigenetic regulation, including DNA methylation and miRNA expression, and an interaction with nuclear proteins has been described. Through these mechanisms, ALK fusion proteins enable a transcriptional programme that drives the pathogenesis of a range of ALK-related malignancies.

## 1. Introduction

Anaplastic lymphoma kinase (ALK) was first successfully cloned in 1994 when it was reported in the context of a fusion protein in cases of anaplastic large cell lymphoma (ALCL) [1]. It was subsequently characterised as a membrane-bound tyrosine kinase expressed during neonatal development of the nervous system although more is known about its roles in disease rather than its normal physiological functions [2]. ALK, as is typical for receptor-protein tyrosine kinases, consists of three domains: an extra-cellular ligand binding domain, a single-transmembrane spanning domain and a cytoplasmic tyrosine kinase domain which is significant for disease pathologies [2]. Indeed, the tyrosine kinase domain of ALK is retained in ALK-containing fusion proteins (See Table 1), resulting in oncogenic tyrosine kinases capable of driving oncogenesis through several different pathways in diverse malignancies. Through the course of this review, these means shall be discussed with an emphasis placed on how Nucleophosmin 1 (NPM1)-ALK (the most studied ALK-fusion protein) mediates oncogenesis through transcriptional regulation. Furthermore, other ALK fusion proteins shall be discussed, such as echinoderm microtubule-associated protein-like 4 (EML4)-ALK which is implicated in non-small cell lung carcinoma (NSCLC). The potential role of the fusion partner to ALK in oncogenesis will also be addressed. As such this review will cover a spectrum of literature, addressing modes of transcriptional regulation and ALK-driven pathologies, as well as ALK-fusion partner proteins and the significance they hold.

## 2. Nucleophosmin 1-Anaplastic Lymphoma Kinase (NPM1-ALK)

**Figure 1:**
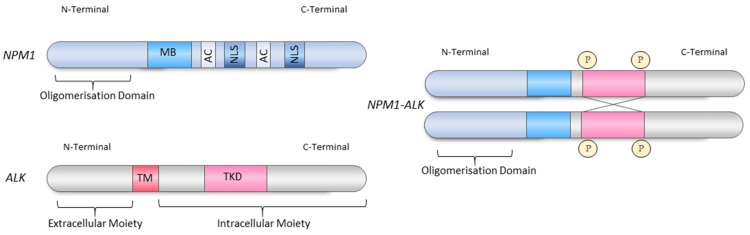
The Nucleophosmin 1 (NPM1)-ALK fusion protein resulting from the t(2;5)(p23;q25)**.** Fusion of the *NPM1* gene on chromosome 5 to the *ALK* gene on chromosome 2 results in the expression of NPM1-ALK, a constitutively activate tyrosine kinase. *NPM1* encodes an oligomerisation domain (residues 1–117), a metal binding domain (MB; residues 104–115), two acidic amino acid clusters (AC: D and E amino acid rich domains which function as acceptor regions for nucleolar targeting signals; residues 120–132 and 161–188) and two nuclear localisation signals (NLS; residues 152–157 and 191–197). *ALK* encodes a Meprin/A5/protein tyrosine phosphatase domain (not shown), a ligand-binding site (residues 391–401) in the extracellular domain, a lipophilic transmembrane region (TM) and an intracellular domain that contains the tyrosine-kinase catalytic domain (TKD).

The t(2;5)(p23;q35) chromosomal translocation associated with ALCL fuses the ALK-encoding intracellular domains on chromosome 2, with the oligomerisation domain-encoding regions of the *NPM1* gene on chromosome 5. This forms a fusion gene that encodes NPM1-ALK; a ligand-independent, intracellular 80 kDa chimeric protein with constitutive tyrosine kinase activity. Through intermolecular trans-phosphorylation, the oligomerised ALK domains are phosphorylated and consequently activated (Figure 1) [3,18]. NPM1-ALK is by far the most common and well described ALK fusion protein detected in patients diagnosed with ALCL (accounting for 70–80% of ALK+ ALCL cases [19]) suggesting either that these two genes are predisposed in some way to translocation events in the precursor cells of ALCL and/or that an NPM1 translocation partner provides a selective advantage to incipient tumour cells over other fusion proteins [3,10,20,21]. Reasons for the latter are not immediately apparent but may be related to the additional nuclear localisation of NPM1-ALK (due to oligomerisation with wild-type NPM1) compared to other fusion proteins, which are restricted to the cytoplasm [10]. It is also important to state the reciprocal fusion product (ALK-NPM1) is not expressed in ALCL so is not pathologically relevant [18].

### 2.1. NPM1-ALK Is a Constitutively Active Tyrosine Kinase Activating a Plethora of Signal Transduction Pathways

**Figure 2:**
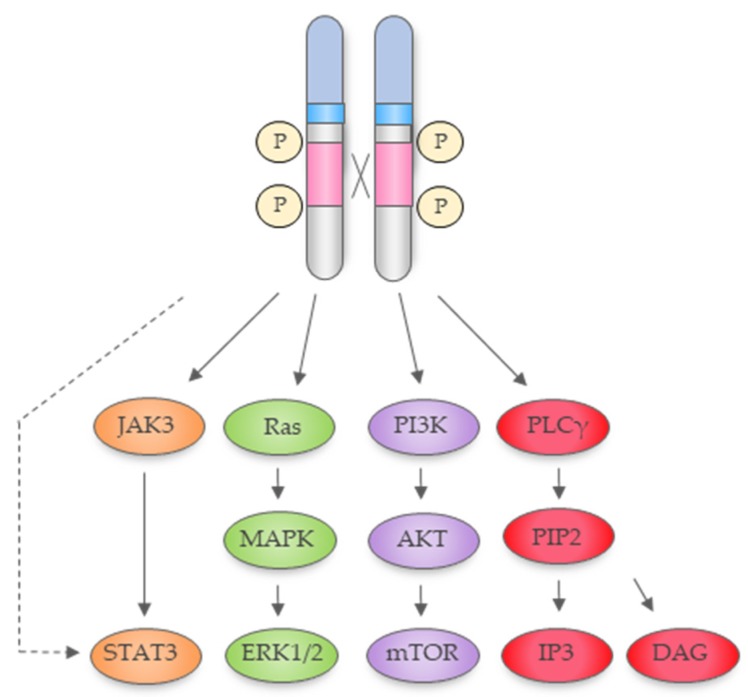
The NPM1-ALK fusion protein signals through the Janus protein tyrosine kinase (JAK)/STAT, Ras/mitogen-activated protein kinases (MAPK), phosphatidylinositol 3-kinase (PI3K)/Akt and phospholipase C gamma (PLC-γ) pathways. Adapted from Trigg et al., 2018 [23].

In the cytoplasm, NPM1-ALK activates a number of interconnected pathways via recruitment of a signalling complex, which results in cellular proliferation, survival and phenotypic changes [22]; the most studied of which are the Ras-extracellular signal-regulated kinase (ERK), Janus kinase (JAK3)-signal transducer and activator of transcription 3 (STAT3), phospholipase C gamma (PLC-γ) and phosphatidylinositol 3-kinase (PI3K)-Akt pathways (Figure 2).

#### 2.1.1. Ras-Extracellular Signal-Regulated Kinase Pathway

Ras proteins (low-weight GTP-binding proteins) hold vital roles in controlling the activity of several signalling pathways which regulate normal cell proliferation [24]. The NPM1-ALK fusion protein is vital in phosphorylating and activating ERK through the activity of the mitogen-activated protein kinases (MAPK) [25,26]. Activation of the ERK1/2 complexes results in the potentiation of a number of other substrates which operate in multiple cell functions including proliferation, survival, migration, cell division and differentiation. In the context of NPM1-ALK+ ALCL, ERK1/2 are known to drive proliferation by promoting cyclin-dependent kinase 4 (CDK4) activity and phosphorylation of retinoblastoma protein, and maintaining viability by positively regulating the expression of anti-apoptotic factors (e.g., Bcl-xL); ERK1 rather than ERK2 holds prominence in maintaining cell viability [27].

#### 2.1.2. The Janus Kinase 3−Signal Transducer and Activator of Transcription 3 Pathway

Cytoplasmic Janus protein tyrosine kinases (JAKs) are essential elements of a variety of pathways which govern cellular survival, proliferation, differentiation and apoptosis [28]. One such JAK, JAK3, is a common upstream activator of the well-characterised transcription factor STAT3 [29]. The oncogenic activity of STAT3 is mediated by NPM1-ALK on several fronts. On a post-translational level, NPM1-ALK positive cell lines express STAT3 phosphorylated on Tyrosine 705 and Serine 727. Furthermore, activated NPM1-ALK induces higher expression of both transcript and protein levels of STAT3. Interestingly, although JAK3 is found to be phosphorylated in NPM1-ALK+ ALCL cells, JAK3 binding and activation is not essential for the activity of STAT3. Moreover, STAT3 activity is seen to be independent of the activity of the proto-oncogene tyrosine-protein kinase Src. This suggests that NPM1-ALK may activate STAT3 directly. Activated nuclear STAT3 has been implicated in maintaining cell survival by controlling the transcription of apoptosis-regulating proteins (e.g., Cyclin D1, Bcl-X, Bcl-X_L_, survivin and c-Myc) thus promoting NPM1-ALK+ ALCL survival [30,31,32].

#### 2.1.3. Phospholipase C Gamma (PLCγ) Pathway

Growth signals frequently result in the activation of PLCγ and PI3K, in turn controlling a broad-range of cellular processes, including cell proliferation, differentiation, survival, shape and migration [33]. Phosphorylated Tyrosine 664 of NPM1-ALK has been identified as a binding site for PLCγ [34]. Through binding specifically to the Src homology 2 (SH2) domains of PLCγ, NPM1-ALK phosphorylates and activates it [35]. Typically, PLCγ has important functions in signal transduction, for example, through the generation of diacylglycerol and inositol triphosphate (IP3)—activators of protein kinase C (PKC). Furthermore, PLCγ activity is reported to be important for DNA synthesis [35]. NPM1-ALK can associate with and phosphorylate other SH2 domain-containing adapter proteins including SHC, Grb2 and Insulin Receptor Substrate-1 (IRS-1) [35]. PLCγ, unlike STAT3, does not appear to be involved in mediating anti-apoptotic responses [35].

#### 2.1.4. Phosphatidylinositol 3-Kinase–Protein kinase B (Akt) Pathway 

The PI3K/Akt signal transduction pathway, like those described above, has been well described for its roles in oncogenic progression. The PI3K/Akt pathway is largely important for regulating cell-cycle progression through controlling the expression of proteins involved in proliferation, such as cyclins and cyclin-dependent kinases [36]. NPM1-ALK recruits the C-terminal SH2 domain of the PI3K p85 subunit. The NPM1-ALK-p85 association is thought to provide sufficient signal for the recruitment of the PI3K p110 catalytic subunit, resulting in PI3K phosphorylating phosphatidylinositol (PI), phosphatidyl 4–phosphate (PI_4_P), and PI_4,5_P_2_. The 3-phosphoinositide products are able to bind to Pleckstrin homology domains (PH) of signalling molecules and to activate various downstream proteins, such as the serine/threonine kinase Akt/PKB [37]. In general, Akt in turn is able to phosphorylate Bcl-2-associated death promoter (BAD), the Bcl2 family member involved in suppressing apoptosis and promoting cellular survival [38]. Furthermore, Akt itself is involved in the inhibition of Caspase-9, activation of nuclear factor kappa-light-chain-enhancer of activated B cells (NFκB) and a reduction in transcription of Fas ligand. Such an anti-apoptotic signal is vital for the molecular pathogenesis of NPM1-ALK+ ALCL [39]. 

#### 2.1.5. Epigenetic Pathways

**Figure 3:**
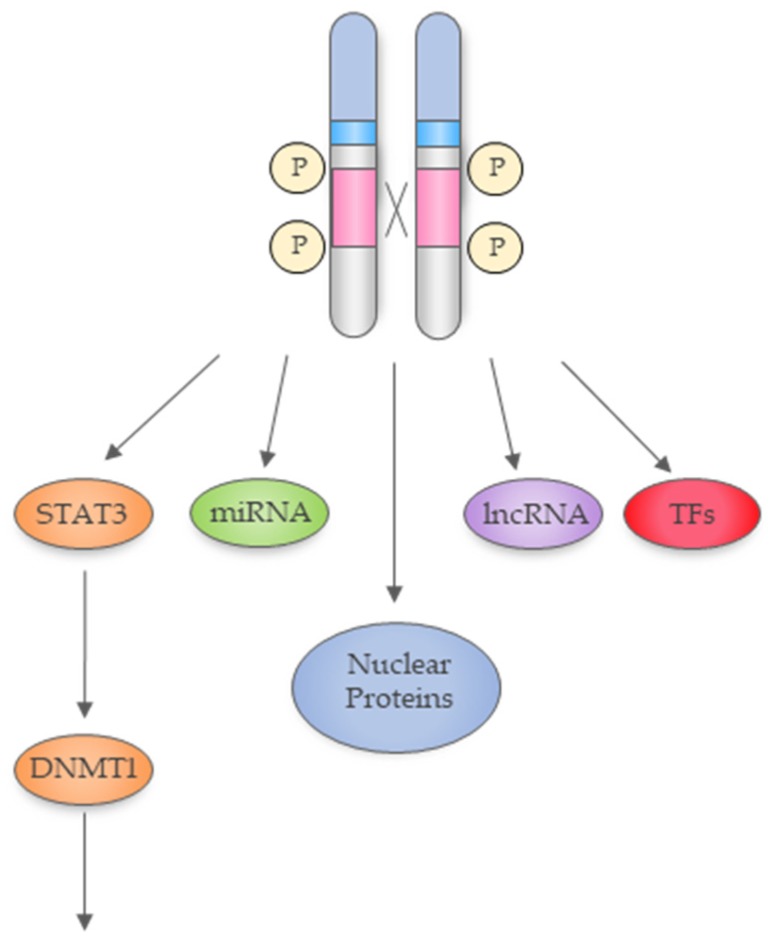
The NPM1-ALK fusion protein drives epigenetic change through DNA methyltransferase (DNMT), miRNA, and transcription factor (TF) expression and through interacting with nuclear proteins.

NPM1-ALK is reported to mediate epigenetic modifications on a number of interlinking fronts, which ultimately regulate gene expression [40]. Firstly, NPM1-ALK-driven transcription factors mediate transcriptional silencing through DNA methylation of gene promoters and enhancer regions [41]. Additionally, NPM1-ALK is linked to modifying the epigenetic landscape through non-coding RNAs such as microRNAs (miRNAs or miRs) [42]. Thirdly, and although less studied, some findings suggest NPM1-ALK plays a role in transcriptional regulation by interacting with nuclear proteins [22,43,44] (Figure 3). 

### 2.2. NPM1-ALK Mediates DNA Methylation

DNA methylation is an epigenetic modification which results in the covalent transfer of a methyl group to the C-5 position of the cytosine ring (hence forming methyl-cytosine) and is mediated by DNA methyltransferases (DNMTs), namely DNMT1, DNMT3a and DNMT3b [45,46]. DNA methylation mainly occurs at CpG dinucleotides and is implicated in many physiological processes, including normal development during embryogenesis, genomic imprinting, X-chromosome inactivation, suppression of repetitive element transcription, gene transcription and transposition [47]. CpG islands are defined as regions of about 500 base pairs in length with a high multimer level of CpGs (more than 55%); they occur in about 60% of all human genes and are usually un-methylated in somatic cells irrespective of their activity. In cancer cells, epigenetic alterations occur that typically comprise of a global loss of methylation and/or locus-specific hypermethylation of CpG islands in promoters [40]. This results in gene silencing of tumour suppressor genes and protein products which regulate cell-cycle progression, signal transduction, DNA repair and oncogene expression [40,48]. Methylation of DNA is thought to sterically impede the binding of transcriptional regulators to the gene either directly or by additional binding by methyl binding proteins, such as methyl CpG binding protein 2 (MeCP2), which are capable of binding to single CpG sites [49,50,51]. Furthermore, methylation prevents binding of the Sp1 transcription factor and additionally impedes the interaction of chromatin with other chromatin modifiers (e.g., polycomb repressive complex [52]). In addition, evidence suggests that DNA methylation directs histone modifications [53].

In the case of NPM1-ALK+ ALCL, NPM1-ALK functions through the transcription factor STAT3, which binds to gene promoters and enhances the binding of DNA methyltransferases [54]. Moreover, Wasik et al. described a relationship between STAT3 and DNMT1, whereby STAT3 induces expression of DNMT1—a key effector of epigenetic silencing [55]. Further driving factors of transcription (including the activator protein 1 (AP1) family member JunB) are suggested as being upstream regulators and are associated with hypo-methylated CpG sites (hypomethylated in ALCL as compared to normal CD3 T cells). Enrichment of AP1 at unmethylated sites might protect them from DNA methylation [41]. JunB binds distinctly to hypomethylated promoters including those of *SERPINA1*, *LYN* and *TLR6*, confirming an important function for AP1 signalling in ALCL as discussed further below [41]. 

For ALK+ ALCL, a number of methylated genes have been identified, many of which are components of the T cell receptor (TCR) signalling pathway, which is therefore silenced. For example, 7 genes involved in the TCR and CTLA-4 pathways show hypermethylation in ALCL patient samples: *CD3*, *CD28*, *CTLA-4*, *LCK*, *GADS*, *SHP1* and *LYP* [41]. Indeed, down-regulation of the TCR has been suggested as a prerequisite for T cell lymphomagenesis [56]. Other genes affected by DNA methylation in ALCL include *p16^INK4A^* [57], *TNF-α* [58], *NFATC1* [59], *IL-2Rγ* (through which Interleukin (IL)-2, -4, -7, 9, -15 and -21 signal) [54] and *BIM* [41]—genes important for cellular proliferation and survival [41,60,61]. 

Conversely, methylation of negative regulators of signalling (such as the phosphatase protein tyrosine phosphatase, non-receptor type 6 [PTPN6 or SHP1]) maintains NPM1-ALK signalling cascades [39]. SHP1 is a tumour suppressor, which functions as a negative regulator of NPM1-ALK and several signal transduction proteins (such as cytokine receptors) by dephosphorylating the receptor itself and/or receptor-associated kinases. SHP1 can also downregulate the activation of STATs, and potentially other signalling pathways driven by NPM1-ALK [34]. Data from ALCL cell lines suggest that STAT5A is another example of an epigenetically silenced protein in ALK+ ALCL. This is achieved through NPM1-ALK-induced STAT3 activity, resulting in CpG Island methylation. STAT5A is a tumour suppressor protein which is capable of reciprocally suppressing *NPM1-ALK* gene expression by selectively binding to the enhancer region [49]. Thus, numerous studies have shown that silencing of tumour suppressor genes by DNA methylation could also play a role in aberrant ALK-induced malignant transformation.

### 2.3. NPM1-ALK Activates microRNAs (MiRNAs; miRs)

Another commonly exploited form of gene regulation is achieved through the dysregulated activity of miRNAs. miRNAs are small, highly conserved non-coding RNAs which function by activating the RNA-induced silencing complex (RISC) against specific mRNA targets [62]. miRNAs function as a guide by specific base-pairing complementary to target mRNA; this complementarity to the 3’-untranslated region determines whether the mRNA target will be silenced by degradation or translational inhibition [63]. 

Aberrantly expressed miRNAs have been associated with many cancers, behaving in either oncogenic (e.g., miR-21, miR-155) or tumour suppressive (e.g., miR-26a, miR-34a) fashions [64]. Several studies, such as that by Liu et al., have investigated the role of miRNAs in ALCL [42,65]. Liu et al. identified 32 miRNAs associated with ALK expression in vitro, noting that ALK positive and ALK negative ALCL show distinct miRNA expression profiles, with 7 distinct miRNAs discerning ALK+ ALCL from ALK− ALCL [66]. These include 5 up-regulated miRNAs (miR-512-3p, miR-886-5p, miR-886-3p, miR-708 and miR-135b) and 2 down-regulated miRNAs (miR-146a and miR-155) [66]. Furthermore, miR-135b has also been implicated in the pathogenesis of ALCL; in particular it has been shown to mediate the NPM1-ALK-induced T-helper 17 (T_h_17) immunophenotype of ALCL [67]. In the context of further tissue-types, roles for the other identified miRNAs include deregulation of apoptosis by miR-886 targeting of BCL2-associated X, apoptosis regulator (BAX; cervical carcinoma) [68], negative regulation of Wnt signalling following miR-708 targeting of transmembrane protein 88 (TMEM88; lung adenocarcinoma) [69], and cell cycle inhibition by miR-512-3p targeting Waf1/Cip1 (Epithelial cell-line models) [66]. The roles of miR-146 and miR-155 are less clear, although miR-146 is known to play a role in cytokine signalling [70], while miR-155 is known to affect various physiological functions, including development of the haematopoietic lineage, differentiation, immunity and inflammation, which have been described in ALK- ALCL immune cell models [65,71].

Another partially overlapping study identified 14 miRNAs that are down-regulated in ALK+ ALCL (in cell lines, murine models and primary patient samples); including the miR-17–92 cluster, miR-101, miR-29c and mir-26a [42]. It is known that the miR-17~92 cluster is transcriptionally regulated by STAT3, and is suggested to be vital for cell viability as it’s inhibition results in cell death in ALK+ ALCL [42,66]. In general, targets of miR101 include: mTOR, MCL1 (an anti-apoptotic protein) and enhancer of Zeste 2 polycomb repressive complex subunit 2 (EZH2; a histone methyltransferase) [42]. In some cancers, epigenetically silenced miRNAs, which have tumour suppressive functions, could serve as therapeutic nodes by restoring their expression and function [63,72]. Although NPM1-ALK evidently has a driving role in moderating differential miRNA expression, for the most part this NPM1-ALK-miRNA mediated transcription mechanism remains to be elucidated. 

The mechanism by which DNA methylation and miRNA expression act in combination to regulate gene expression is often subverted to facilitate malignant transformation and can result in the development of blood cancers. This is exemplified by the relationship between DNMT1 and miR-150. As described above, NPM1-ALK is vital in driving STAT3 activation, which in turn induces DNMT1 to enhance gene repression by DNA hypermethylation [55,73]. MiRNA-150 is predominantly expressed in haematopoietic cells of the spleen and lymph nodes and is known to contribute to basal cell functions and to lead to the development of haematopoietic malignancies when absent [74,75]. Accordingly, ectopic expression of miR-150 inhibits cellular proliferation by down-regulating expression of MYB and as such prevents S-phase entry of NPM1-ALK+ cells in vitro [76].

### 2.4. Long Non-Coding RNAs Are Expressed in ALCL

Additional non-coding RNAs such as long non-coding RNAs have also been associated with regulating transcription [77]. lncRNAs have been implicated in a broad range of developmental processes and pathologies, but the precise mechanisms through which they exert their function is still quite poorly understood [78]. However, it is known that lncRNAs regulate target gene expression through modulation of DNA methylation at CpG dinucleotides in addition to direct interaction with transcription factors to affect their activity and subcellular localisation. Moreover, lncRNAs also have roles in post-transcriptional regulation at the levels of mRNA processing, stability and translation regulation [78,79,80,81].

Of relevance to this review, a number of lncRNAs have notably been associated with a variety of cancers and have been determined to play pivotal roles [82]. Furthermore, certain lncRNAs have been associated with disease progression, diagnosis and even act as therapeutic targets [83]. Examples include: prostate cancer-associated transcript 1 (PCAT-1), which promotes proliferation and is a target of polycomb repressive complex 2 (PRC2), whose regulation has been linked with prostate cancer [83]; antisense non-coding RNA in the INK4 Locus (ANRIL) which represses the tumour suppressors p16^INK4A^ and INK4b/p15^INK4B^, and which is upregulated in prostate cancer [84]; and HOX antisense intergenic RNA (HOTAIR), whose overexpression is associated with poor prognosis in breast, liver, colorectal, gastrointestinal and pancreatic cancers, and has been proposed to increase tumour invasiveness and metastasis [85]. 

A number of up-regulated lncRNAs have been detected in ALCL, including BMS1 pseudogene 20 (BMS1P20), long intergenic non-protein coding RNA 1012 (LINC01012), Mir503HG, RNG144-AS1 and calcium voltage-gated channel subunit alpha1 G (CACNA1G-AS) [82]. In particular, LINC01013 has been associated with a role in ALCL invasion through activation of the snail pathway [82]. Similarly, in another study, compared to normal T-lymphocytes, 83 lncRNAs were expressed in ALCL patient samples—one of which, BlackMamba, was only seen in ALK-ALCL cases [79]. However, the significance of these lncRNAs to the lymphomagenic process and the pathology of ALCL for the most part remains to be determined.

### 2.5. NPM1-ALK Driven Transcription Factors

NPM1-ALK is well described in driving expression of both basic leucine zipper (bZIP) and basic helix-loop-helix (HLH) transcription factors [80,81,86,87,88]. bZIP transcription factors are characterised by a conserved bZIP region which enables DNA binding and include the AP1 complexes which have been extensively characterised in ALK+ ALCL [80]. AP1 is a sequence-specific DNA binding factor formed as a dimeric complex with various members of the Jun (cJUN, JUNB, JUND), Fos (cFOS, FRA1, FRA2) and activation transcription factor (ATF) family [81]. For ALK+ ALCL, expression of both basic leucine zipper ATF-like transcription factor (BATF) and BATF3 is upregulated; both of these proteins bind classical AP1 motifs and interact with other AP1 transcription factors in ALCL [80]. 

AP1 complexes regulate the expression of proteins involved in cellular differentiation, proliferation and survival and can therefore potentiate malignant transformation when dysregulated in ALCL and other malignancies [81]. BATFs additionally interact with interferon regulatory factor 4 (IRF4; known to drive MYC expression) to cooperatively enhance DNA binding to so-called AP1-IRF composite elements in immune cells [86]. NPM1-ALK also induces AP1 transcription factors by the signalling pathways discussed above (e.g. IRS-1, SHC and PLCγ) [87]. Furthermore, it has been demonstrated that catalytically active NPM1-ALK is required for AP1 transcriptional activity and that cJUN is activated via the NPM1-ALK-dependent c-Jun N-Terminal Kinase (JNK) pathway [88]. cJUN and JUNB furthermore have roles in promoting cell cycle progression through regulation of cell-cycle checkpoints [88].

### 2.6. NPM1-ALK Interacts with Nuclear Proteins 

As NPM1-ALK has the ability to shuttle into the nucleus via its interactions with wild-type NPM1, it can interact with nuclear proteins and alter their function as eluded to above. Whether this activity is due to NPM1-ALK itself or due to disruption of wild type NPM1 function is not completely known. 

Using a proteomic approach, Galietta et al. (2007) identified a number of RNA/DNA-binding proteins which were found to co-immunoprecipitate with NPM1-ALK, including polypyrimidine tract binding protein-associated splicing factor (PSF) [44]. PSF is a multi-functional nuclear protein involved in a plethora of diverse functions: pre-mRNA splicing, gene transcription, DNA repair, DNA recombination and cytoplasmic mRNA stability [44]. PSF is directly phosphorylated by NPM1-ALK at tyrosine 293—this phosphorylation site is critical for the physical association between NPM1-ALK and PSF [44]. Functionally, this phosphorylation event alters PSF’s subcellular localisation from nuclear to cytoplasmic (whilst a proportion remains in the nucleus). Additionally, this phosphorylation event increases PSF’s RNA binding ability and up-regulates transcriptional repression activities [44,89]. However, it is important to note that PSF is localised solely in the nucleus in non-ALK-fusion protein-expressing cells [44].

Four additional nuclear RNA/DNA binding proteins that associate with NPM1-ALK have been described: the nuclear RNA-binding protein 54 kDa (p54^nrb^), translocated in liposarcomas (FUS/TLS), expressed in Ewing sarcoma (EWS) and nucleolin [44]. These data support the findings of Crockett et al. (2004) who similarly reported a number of both cytoplasmic and nuclear proteins to co-immunoprecipitate with NPM1-ALK using proteomic approaches [22]. In addition to several proteins which had previously been reported to interact with NPM1-ALK (PI3K, PLCy1, JAK2, JAK3, STAT3 and IRS), a number of proteins that bind to components of the nuclear matrix were also identified: similar to Nucleophosmin (NPM), Centromere protein F, CASK-Interacting Protein 1, CDC14 (a protein which is associated with the centrosome structure), SN24 HUMAN, Nuclear protein GRB1 (BRG1), Cyclophilin D, Nucleoporin NUP98, Grb7v, Nuclear Mitotic Apparatus Protein, and Chromosome Condensation Protein G—although this may be reflective of proteins which interact through the NPM1 retained component of the NPM1-ALK fusion protein. The caveat of these papers is that both sets of experiments were undertaken using total cell lysates, as such it is not possible to determine the localisation of the NPM1-ALK-protein interaction. Furthermore, the immunoprecipitation for the second Crockett et al. data set was performed with an NPM1 N-terminal antibody which would also immunoprecipitate the endogenous wild-type NPM1 [22]; thus it cannot be excluded that the proteins co-immunoprecipitated in this data set may include proteins that interact solely with wild-type NPM1, but not with NPM1-ALK directly. 

Nuclear-interacting partner of ALK (NIPA) is a 60 kDa downstream target of NPM1-ALK which contains a nuclear translocation signal in its C terminus. NIPA interacts with NPM1-ALK in a kinase-dependent manner and is phosphorylated by NPM1-ALK on tyrosine residues. Furthermore, NIPA is phosphorylated by an unidentified serine kinase at residue S354 in cells that exogenously express kinase-active ALK fusion proteins [43]. 

Thus, the literature supports the idea that the NPM1-ALK fusion protein can interact with nuclear proteins. Ultimately, NPM1-ALK must oligomerise to be active and transformative [3,10], and this capacity is shared among all ALK fusion partner genes. While the oligomerisation domains of NPM1 are critical for the transforming capacity of NPM1-ALK, there are other aspects of NPM1 which could contribute to the pathogenic activity of the NPM1-ALK fusion protein.

## 3. NPM1 as an ALK-Translocation Fusion Partner

### 3.1. Known Functions of Nucleophosmin 1

NPM1 is a ubiquitously expressed, multifunctional, nucleolar shuttle protein responsible for transporting proteins between the nucleus and the cytoplasm—it is attributed to both oncogenic and tumour-suppressive functions, with additional physiological roles in ribosome biogenesis [90,91], mRNA processing [92], chromatin remodelling [93], cell growth and proliferation, and regulating apoptosis [94]. The NPM histone chaperone family, of which NPM1 is a member, consists of three conserved structural motifs: an N-terminal core domain, an acidic domain, and a less conserved C-terminus (mostly associated with a nuclear localisation signal) [94,95]. The N-terminal core pentamerisation domain (residues 16-118) facilitates NPM1 oligomerisation, interactions with other (namely nucleolar partner) proteins and contains two putative export signals [94,96]. This facilitates NPM1’s most well-described function—its nucleocytoplasmic shuttling role, whereby NPM1 exports ribosomal protein L5 to the nucleolus [90]. A number of other proteins have been described which are also transported to the nucleolus by NPM1; these include Rev, Rex, Tat and p120 [90,94,97,98,99,100,101,102]. 

The post-translationally modified central aspartic and glutamic-rich acidic regions of NPM1 are known to be involved in the electrostatic binding of histones H1, H2A, H2B, H3 and H4 [96,103,104]. Nucleophosmin family proteins are thought of as “storage platforms” or “sinks” whereby the proteins store histones for an extended period of time before transfer onto DNA takes place [105]. NPM1 binds to H3-H4 tetramers (preferentially) but also to H2A-H2B dimers through the A2 acidic stretch [106]. Through this activity, NPM1 can assemble nucleosomes and, in doing so, regulate DNA replication, recombination, transcription and repair. More so, NPM1 interacts with a plethora of proteins involved in the above processes, including retinoblastoma and c-Myc [107,108]. Finally, the C-terminus is characterised by basic, positively charged amino acids followed by a sequence of aromatic residues, which facilitates the binding of NPM1 to nucleic acids and ATP, and also constitutes an atypical nucleolar localisation signal [94,109,110]. 

NPM1 regulates apoptosis through interactions with p14^ARF^, MDM2 and p53 [94,109,111]. In the absence of cellular stress, p14^ARF^ dimerises with NPM1, allowing MDM2 to target p53 for proteasomal degradation thereby maintaining cell survival. Under stress, p14^ARF^ dissociates from NPM1 resulting in the sequestration of MDM2, stabilisation and activation of p53 and consequential induction and potentiation of apoptosis [94]. Hence, by directing p14^ARF^ to the nucleolus and preventing the inhibition of MDM2, NPM1 regulates cell fate in a p53-dependent manner [112]. Additionally, NPM1 has been suggested to prevent the translocation of p53 from the nucleus to the mitochondria, thereby inhibiting cytochrome c release [94,113]. NPM1 (phosphorylated on threonine 199) has also been implicated in homologous recombination [94,114]. Following DNA double-strand break, NPM1 binds to chromatin and co-localises with other DNA repair proteins, such as BRCA1 and gamma- histone H2AX (γH2AX), therefore playing a role in DNA repair [112]. 

### 3.2. Structure of Nucleophosmin 1

The crystal structure of the core domain of NPM1 has been determined as composed of eight anti-parallel beta-sheets [112]. Monomers assemble into ‘donut-shaped’ homo-pentamers with an asymmetric, negatively charged residue isolated to one side of the oligomers [112]. The pentamers dimerise in a ‘head-to-head’ fashion, forming a decamer through a single monomer contact of the other pentamer [112]. This facilitates structural plasticity at the pentamer-pentamer interface through post-translational modifications, namely phosphorylation [115]. Such events modulate the monomer-pentamer equilibrium of the decamer and have been linked to the regulation of NPM1 localisation and function [18,94].

### 3.3. The Roles of the Retained NPM1 Domains in the NPM1-ALK Fusion Protein

Critically, despite wild-type ALK expression being typically restricted to neural tissues, expression of the NPM1-ALK fusion protein ectopically occurs in NPM1-ALK+ tumour cells via the NPM1 promoter as a result of the t2;5(p23;q25) translocation [3]. Through the retained oligomerisation domain of NPM1 in the NPM1-ALK fusion protein, constitutive tyrosine kinase activation results (independently of ligand stimulation) due to intermolecular trans-phosphorylation, leading to phosphorylation of substrate proteins [3,18]. Additionally, hetero-oligomerisation of NPM1-ALK with wild type NPM1 leads to the nuclear and cytoplasmic localisation of the NPM-ALK protein [3,18]. As described, NPM1 contains two nuclear localisation sequences (NLS) in the N-terminus which mediate its nuclear localisation and activity [3]. Whilst the NLS are not retained in the NPM1-ALK fusion protein, nuclear localisation of NPM1-ALK is achieved through binding to endogenous NPM1 encoded by the remaining intact allele via the oligomerisation domain, which is retained in the fusion protein leading to accumulation within the nucleolus [43]. Whether nuclear/nucleolar NPM-ALK has any significant contributory role in disease pathogenesis continues to be a focus of ongoing research.

While an intact NPM1 segment is essential for the transforming capacity of NPM1-ALK, restriction of ALK expression to the cytoplasmic compartment is sufficient for transformation [3,10] but this does not exclude the possibility that nuclear NPM1-ALK may also contribute to oncogenesis in ALCL cells. To test this hypothesis, Ceccon et al. (2016) exogenously expressed a fusion protein of full-length NPM1 juxtaposed to the ALK domain (NPMtot-ALK) in 293T and BaF3 cells, which showed exclusively nuclear/nucleolar localisation. Unlike NPM1-ALK, NPMtot-ALK was unable to transform these cells, although there was also no demonstration that this non-physiological protein was oligomerisation-competent and capable of auto-activation [21]. Also in this study, the authors present evidence that NPM1-ALK is only phosphorylated and active in the cytoplasmic compartment of ALCL cells, reasoning that nuclear NPM1-ALK is inactive due to heterodimerisation with wild-type NPM1, acting to sequester NPM1-ALK and prevent excessive signalling [21]. This is in marked contrast to the results of Hwang et al. (2017), which show detection of phosphorylated NPM1-ALK in both the nuclear and cytoplasmic compartments of ALCL cells [116], as well as previous research which demonstrated that NPM1-ALK forms and is catalytically active in higher-order oligomeric complexes, consistent with the finding that wildtype NPM1 forms pentameric oligomers [3,112]. Thus, it remains unclear exactly what contribution nuclear NPM1-ALK makes towards the pathogenesis of ALCL. Nevertheless, the NPM1 portion of the fusion protein is critical for NPM1-ALK mediated oncogenic activity, whereby NPM1-ALK mutant proteins lacking overlapping portions of the NPM1 segment are unable to form complexes, lack kinase activity and are unable to transform cells [3].

### 3.4. Is Heterozygous Loss of NPM1 a Key Factor Driving Lymphomagenesis?

The t(2;5)(p23;q25) translocation not only results in the formation of NPM1-ALK but also halves the expression of wild-type NPM1—a protein with a plethora of vital functions, as described above. NPM1 haploinsufficiency contributes to the development of some cancers such as AML, but preliminary findings in murine models suggest that it does not contribute to the development of ALCL. However, these data cannot easily be extrapolated to the pathogenesis of ALK+ ALCL in humans due to non-physiological expression of NPM1-ALK, differences in T-cell development between mouse and human, and the importance of the correct cell of origin for transformation in the pathogenesis of ALCL [56,111,117].

## 4. Other ALK Fusion Proteins Are Causative of Cancer 

NPM1 is not the only ALK fusion partner that has been associated with cancer. ALK fusion proteins have also been described in NSCLC, diffuse large B-cell lymphoma (DLBCL), inflammatory myofibroblastic tumour (IMT), and to a lesser extent oesophageal squamous cell carcinoma (ESCC), renal medulla carcinoma (RMC), renal cell carcinoma (RCC), serous ovarian carcinoma (SOC), and breast and colon cancer [18,118]. EML4-ALK is a notable example, due to its relative predominance in NSCLC [119]. Recently, a novel isoform of ALK (ALK^ATI^) (a truncated isoform, driven from an alternative transcription initiation (ATI) site in intron 19, encoding only the intracellular domain of ALK) has been described as being expressed in ~11% of melanomas and sporadically in other human cancers [120,121].

### 4.1. Do other ALK Fusion Proteins Have a Role in the Nucleus?

Unlike NPM1-ALK, other ALK fusion proteins (e.g., TPM3-ALK, ATIC-ALK, CLTC-ALK and TFG_S/L_-ALK) do not localise within the nucleus; 15–25% of ALK positive ALCL do not exhibit immunohistochemical (IHC) staining patterns of ALK in the nucleus with expression restricted instead to the cytoplasm [18]. All ALK translocation breakpoints are exclusively located in the intron flanked by exons 16 and 17 of *ALK*, with exons 17–26 encoding the intracellular domains of ALK. Therefore, each translocation generates a unique fusion product consisting of the 5’ partner fused to the 3’ intracellular ALK tyrosine kinase domain—all fusion proteins contain exactly the same 563 amino acid sequence which comprises the intracellular region of the ALK protein [18]. It might be expected that all cytoplasm-localised ALK fusion proteins activate similar signalling pathways that may or may not be compromised by the nature of the fusion partner. Despite this, it is clear from immunohistochemical analysis that the majority do not access the nucleus, and therefore differ in this manner from NPM1-ALK [18,122]. ALK^ATI^ (as described above) is present in both the cytoplasm and the nucleus, where it can self-interact, resulting in autophosphorylation and kinase activity [120]; however, the mechanism by which this occurs remains to be fully elucidated. Previous studies have indicated a role for NIPA in translocating X-ALK fusion proteins to the nucleus, and it is possible that others proteins may also be involved in this activity [43]. 

### 4.2. EML4 Is an ALK-Translocation Fusion Partner

Genetic mutations in the epidermal growth factor receptor gene (EGFR; 15/58 cases in Japan and 1/61 cases from the USA) and gene rearrangements which result in the fusion of *EML4* with *ALK* generating the fusion protein EML4-ALK (3–7% of NSCLC cases; 6.7% of Japanese cases) are, except in rare cases, mutually exclusive [119]. This somatic rearrangement was first identified in a small cohort of Japanese patients with NSCLC [4]. It arises from the inversion of the short arm of chromosome 2 (Inv(2))(p21p23)), and results in the expression of a chimeric tyrosine kinase with an N-terminal EML4 moiety juxtaposed to the C-terminal kinase domain of ALK [19]. Notably, various EML4-ALK variants are found to possess different truncations of EML4 while maintaining the same cytoplasmic tyrosine kinase domain of ALK [4].

Since wild-type ALK expression is typically restricted to neonatal neural tissues, it is therefore not endogenously expressed in adult lung tissue. Thus, in the context of EML4-ALK+ NSCLC, ectopic expression of the EML4-ALK fusion protein is conferred by the EML4 gene promoter as the 5’ fusion partner gene [123]. Interestingly, EML4-ALK has also been reported in breast and colorectal cancer but its role in these other cancers has not been as extensively characterised as for NSCLC [118,124]. In addition to EML4-ALK, other oncogenic ALK fusion proteins such as TFG-ALK and KIF5B-ALK have also been identified in NSCLC and described to have oncogenic functions [7,12]. 

#### 4.2.1. Structure and Function of the EML Protein Family

Microtubules are components of the cytoskeleton that are present throughout the cytoplasm and exist as polymers composed of α and β tubulin heterodimers [125]. They are essential in providing mechanical support for cells enabling cell division, intracellular movement and cell motility. The dynamics of microtubules are regulated by microtubule-associated proteins (MAPs) that are generally classified into two types: type 1 (including MAP1) and type 2 (including MAP2 and MAP4). MAP2, cytoskeleton-associated protein 2 (CKAP2) and Tau are involved in stabilising microtubule networks [126], whereas stathmin and katanin are involved in destabilising microtubule networks [127,128].

Echinoderm microtubule-associated protein (EMAP), first identified in the sea urchin *Echinoidea,* is the founding member of the EMAP-like (EML) family of proteins [129]. EML proteins associate with microtubules and are involved in the regulation of microtubule assembly during mitosis. There are 6 different EML proteins described in humans—EML1-6 [130]—which are sub-categorised based on their protein domain structure. EML1 to EML4 contain a C-terminal hydrophobic EML protein domain (HELP), an N-terminal coiled domain and variable tryptophan-aspartic acid (WD) repeats which together contribute to interactions with microtubules [131,132]. A structural study revealed that the N-terminal coiled region of EML2 and EML4 is required for trimeric oligomerisation, and the region was therefore aptly named the trimerisation domain (TD) [133]. 

EML4 is also a microtubule stabilising protein and is essential for cell proliferation and survival [134,135]. Since it is associated with microtubule function, it is expressed in most cell types [134]. Expression analysis of EML4 in normal lung tissues identified EML4 transcripts to be expressed in alveolar macrophages and also in epithelial type II cells, with higher levels in alveolar macrophages [136]. 

#### 4.2.2. EML4-ALK Variants 

In NSCLC, 15 distinct variants of EML4-ALK have been identified, with some variants being expressed as multiple isoforms [137,138,139]. The most common variants are ELM4-ALK-1, -2, -3a and -3b, collectively constituting ~90% of all cases [140]. All variants include the kinase domain of ALK, encoded by exons 20–29, but differ in size based on the EML4 breakage point. Furthermore, all variants of EML4 contain the trimerisation domain (TD), which is required to activate ALK through oligomerisation and autophosphorylation [133]. EML4-ALK variants 3a/b and 5a/b lack the C-terminal TAPE domain of EML4 completely. The shortest variants are EML4-ALK 5a/b which, despite lacking the TAPE domain, possess transforming activity due to the presence of the TD domain [141]. Loss of the globular domain in EML4-ALK results in a relatively unstable fusion protein which recruits HSP90 [137]. Therefore, EML4-ALK variant 1 is sensitive to HSP90 inhibitor treatment and clinical trials in NSCLC patients using these inhibitors have shown promising results [142]. 

Current findings suggest that different EML4-ALK variants might have varying biological and clinical significance in NSCLC. A recent clinical study described EML4-ALK variants with different frequencies of ALK resistance mutation developing upon treatment with ALK tyrosine kinase inhibitors (TKIs) [143]. When treated with the 3^rd^ generation ALK inhibitor Lorlatinib (PF-6463922), ALK resistance mutations were more commonly found in patients carrying EML4-ALK variant 3 as compared to variant 1 patients, but variant 3 was associated with a significantly longer progression-free survival than variant 1 [143]. Similarly, Woo et al. conducted a study on EML4-ALK-positive patients who were treated with an ALK tyrosine kinase inhibitor (ALK TKI) to identify whether variants were associated with different treatment responses [144]. Variant 1-expressing patients responded better than variants 3a/b and it was also shown in in vitro studies that cells expressing variants 1 or 2 are more sensitive towards treatment than cells expressing variants 3a or 5a. Furthermore, the study indicated that the stability of the variants is determined by the EML4 fused region, which might be due to the presence of the TAPE domain in variant 1 and the lack of this domain in variant 3 [132,144]. Thus, a better understanding of EML4-ALK variants’ biological functions and their downstream pathways will enhance therapeutic potential in EML4-ALK positive NSCLC.

#### 4.2.3. Does Localisation of EML4-ALK Affect Its Function?

EML proteins generally associate with microtubules by binding via their TD region and TAPE domains [133]. Localisation studies of EML4-ALK variants 1 and 3 a/b in H2228 and H3122 patient-derived NSCLC cell lines highlight that variant 1 is localized in the cytoplasm, whereas variants 3a/b are localised to microtubules similar to full length EML4 [132,133]. In HeLa and NIH3T3 cell lines, overexpression of variants 1, 2 and 5 are shown to have a cytoplasmic localisation [133,145]. Whereas exogenously expressed variant 3 localises to the microtubules in HeLa cells [133], overexpression of variant 3 in NIH3T3 cells shows both a nuclear and cytoplasmic distribution [145]. However, it should be noted that overexpression of an exogenous protein above physiological levels can affect its subcellular distribution and therefore caution should be taken when interpreting these results. This differential localisation of EML4-ALK fusion variants might direct distinct downstream signalling pathways. 

#### 4.2.4. EML4-ALK Mediated Signalling

Similar to NPM1-ALK, EML4-ALK drives the constitutive activation of a plethora of downstream intracellular pathways (e.g., Ras/ERK, PI3K/Akt and JAK/STAT pathways) that cumulatively result in increased proliferation and reduced apoptosis of transformed cells [145]. Moreover, there are emerging studies that corroborate the role of epigenetics in EML4-ALK NSCLC [146,147]. A recent study identified a miR-1253 as a biomarker in EML4-ALK NSCLC, distinguishing it from other types of NSCLC. However, the role of miR-1253 in EML4-ALK NSCLC has not yet been fully elucidated [146]. Another study reports that the lysine residue, K1610 in the tyrosine kinase domain of EML4-ALK is likely methylated by the lysine methyltransferase SMYD2 to modulate EML4-ALK kinase activity and signalling [147]. Therefore, this study suggests that post-translational methylation of EML4-ALK may mediate its oncogenic effects in NSCLC. 

Zhang et al. utilised phosphoproteomics and RNA interference screens to study ALK signalling in EML4-ALK positive NSCLC cell lines [148]. This study identified two scaffolding proteins (FRS2 and CC2D1A) which could sensitise cells to ALK inhibitors, specifically Crizotinib (Xalkori) and Alectinib (Alecensa). Such findings could help to improve ALK inhibitor efficacy for some patients in the future [148]. Finally, recent studies found that high PD-L1 expression is associated with EML4-ALK fusion protein expression in NSCLC [149]. EML4-ALK mediated upregulation of PD-L1 provides a strategy to block PD-L1 as a treatment option in ALK-tyrosine kinase inhibitor resistant NSCLC [149]. These data highlight that an improved understanding of EML4-ALK-induced signalling pathways could help to improve the future treatment of EML4-ALK+ NSCLC in the clinic.

## 5. Expression of Full-Length ALK in Other Cancers

ALK drives other cancers through mechanisms independent of fusion proteins entirely. The pathobiology of ALK amplification and ALK driver mutations for instance is well studied in the context of neuroblastoma [150]. In this context, wild-type (full length) ALK is susceptible to single-base pair missense mutations in key regulatory regions of the tyrosine kinase domain, thereby perpetuating ligand-independent signalling through disrupting the auto-inhibited allostery of the active-site [151]—namely three key ‘hot spot’ mutations which account for 85% of ALK mutations in neuroblastoma; R1275, F1174, and F1245 [152]. Such activating mutations have also since been implicated in additional cancer types, including anaplastic thyroid cancer [153], rhabdomyosarcoma, primitive neuroectodermal tumour and osteosarcoma [154]. Furthermore, an additional mechanism of ALK activation in neuroblastoma is attributable to gene amplification, which results in increased protein expression and constitutive catalytic activity [155]. While gene amplification and point mutations have not been described in driving ALCL nor NSCLC, owing to the absence of wild-type ALK expression, these mechanisms have been described in relation to the fusion proteins as a means of ALK TKI resistance [156,157].

These findings raise the hypothesis that ALK-mediated oncogenesis may be dependent on only two common factors. Firstly, there must be the capacity for constitutive ALK activation (which can be conferred through autophosphorylation as a consequence of oligomerization in the case of ALK fusion proteins, or to oligomerize through ligand independent stimulation in the case of oncogenic ALK mutant proteins). Secondly, there must be a mechanism for expression of ALK (which may be ectopically driven via a translocation partner gene promoter in the case of fusion proteins in ALCL and NSCLC, or endogenously driven in the case of mutant ALK species in neuroblastoma). Indeed, detection of ALK expression is critically important for both diagnosis (using fluorescence in situ hybridization [FISH], next generation sequencing, or immunohistochemistry) and the direction of appropriate treatment regimens [158,159,160,161,162,163,164,165,166].

## 6. Conclusions

It is evident that several interconnected gene regulatory systems mediated by NPM1-ALK contribute to the pathogenesis of ALK+ ALCL. It is also clear that a large number of ALK fusion proteins are prevalent in driving a diverse assortment of malignancies, although due to the relative rarity of these malignancies, many remain poorly understood. By further understanding the roles of ALK fusion proteins and their downstream effectors in the nucleus and by gaining insights into the epigenetic mechanisms contributing to their oncogenic activities, such findings could offer potential relevance for clinical therapy by providing novel targets for the treatment of ALK+ malignancies. More so, by comprehending the molecular similarities throughout the diverse miscellanea of ALK fusion proteins, potential therapeutic targets may present themselves, and thus improve the prognosis of ALK-related cancers.

## Figures and Tables

**Table 1:** An assortment of anaplastic lymphoma kinase (ALK) fusion proteins have been described in anaplastic large cell lymphoma (ALCL) and non-small cell lung carcinoma (NSCLC).

ALK Fusion Proteins	Gene Name	Translocation	Localisation	Cancer Type	References
NPM1-ALK	Nucleophosmin 1	t(2;5)(p23;q35)	Cytoplasm, Nucleus, Nucleolus	ALCL	[3]
EML4-ALK	Echinoderm Microtubule Associated Protein-Like 4)	inv(2)(p21p23))	cytoplasm	NSCLC	[4]
ATIC-ALK	5-Aminoimidazole-4-carboxamide ribonucleotide formyltransferase/IMP cyclohydrolase	inv(2)(p23q35)	cytoplasm	ALCL	[5]
CLTC-ALK	Clathrin heavy chain	t(2;17)(p23;q23)	cytoplasm	ALCL	[6]
TPM3-ALK	Tropomyosin 3	t(1;2)(q25;p23)	cytoplasm	ALCL	[7]
TPM4-ALK	Tropomyosin 4	t(2;19)(p23;p13)	cytoplasm	ALCL	[8]
TFG-ALK	TRK-fused gene	t(2;3)(p23;q21)	cytoplasm	ALCL	[7]
TRAF1-ALK	TNF receptor associated factor 1	t(2;9)(p23;q33)	cytoplasm	ALCL	[9]
RNF213-ALK	Ring finger protein 213	t(2;17)(p23;q25)	cytoplasm	ALCL	[6]
MYH9-ALK	Myosin heavy chain 9	t(2;22)(p23;q11)	cytoplasm	ALCL	[10]
MSN-ALK	Moesin	t(X;22)(q11;p23)	cytoplasm	ALCL	[7]
EEF1G-ALK	Eukaryotic translation elongation factor 1 gamma	t(2;11)(p23; q12.3)	cytoplasm	ALCL	[11]
KIF5B-ALK	Kinesin family member 5B	t(2;10)(p23;p11)	cytoplasm	NSCLC	[12]
KLC1-ALK	Kinesin light chain 1	t(2;14)(p23;q32)	cytoplasm	NSCLC	[13]
STRN-ALK	Striatin	del(2)(p22p23)	cytoplasm	NSCLC	[14]
PTPN3-ALK	protein tyrosine phosphatase, non-receptor type 3	t(2;9)(p23;q31)	cytoplasm	NSCLC	[15]
DCTN1-ALK	Dynactin subunit 1	t(2;2)(p13;p23)	cytoplasm	NSCLC	[16]
GCC2-ALK	GRIP and coiled-coil domain-containing protein 2	t(2;2)(p23;q12)	cytoplasm	NSCLC	[17]
